# Multifunctional Near-Infrared-Responsive Silk Fibroin Nanomedicine for Tumor Treatment and Imaging

**DOI:** 10.3390/ma19153359

**Published:** 2026-08-06

**Authors:** Die Xu, Shanshan He, Jingzhu Xing, Li Hao, Zhijun Zhang, Miao Su

**Affiliations:** 1 College of Textile Science and Engineering (International Silk Institute), Zhejiang Sci-Tech University, Hangzhou 310018, China; 2 Shengzhou Innovation Research Institute of Zhejiang Sci-Tech University, Shengzhou 312400, China; 3 Department of Chemistry, School of Chemistry and Chemical Engineering, Zhejiang Sci-Tech University, Hangzhou 310018, China; 4 Zhejiang Provincial Key Laboratory of Silk and Silk Protein New Materials, Hangzhou 310018, China

**Keywords:** silk fibroin, phototherapy, chemodynamic therapy, synergistic therapy, fluorescence imaging

## Abstract

**Highlights:**

- A facile adsorption strategy constructs NIR-responsive silk fibroin nanoparticles co-loaded with ICG and Fe^3+^ for activatable theranostics.
- The tumor microenvironment disrupts ICG–Fe^3+^ coordination, switching on long-lasting fluorescence imaging over 48 h.
- Molecular docking and DFT calculations reveal a hydrogen-bond-driven ICG loading and LMCT-based fluorescence quenching mechanism.
- High photothermal conversion efficiency (48.7%) enables PTT-boosted CDT, while CDT-produced ^1^O_2_ amplifies PDT for potent triple synergy.
- Complete 4T1 tumor eradication and excellent biocompatibility highlight the translational potential of this silk-based nanoplatform.

**Abstract:**

The complexity and heterogeneity of tumors make monotherapy inadequate for effective tumor elimination, highlighting the urgent need for multifunctional synergistic therapeutic strategies. In this study, a near-infrared (NIR)-responsive multimodal therapeutic nanoplatform (FSINPs) was constructed by simple adsorption of indocyanine green (ICG) and Fe^3+^ onto silk fibroin nanoparticles. Molecular docking showed that ICG binds stably to silk fibroin mainly via hydrogen bonds. Density functional theory (DFT) calculations predicted that Fe^3+^ strongly coordinates with the sulfonate groups of ICG and quenches ICG fluorescence via intermolecular charge transfer. Under conditions mimicking the acidic and high-glutathione tumor microenvironment, the ICG-Fe^3+^ coordination is disrupted, leading to fluorescence recovery of FSINPs. Fe^3+^ catalyzes the Fenton reaction to generate hydroxyl radicals (·OH), thereby achieving chemodynamic therapy (CDT). Upon 808 nm laser irradiation, ICG acts as a dual photosensitizer capable of both photothermal therapy (PTT) and photodynamic therapy (PDT), generating local hyperthermia with a photothermal conversion efficiency as high as 48.7% and producing singlet oxygen (^1^O_2_). The photothermal effect facilitates ·OH production, and CDT enhances photodynamic efficacy. The synergistic action of the three therapeutic modalities results in potent light-activated cytotoxicity toward tumor cells. In vivo experiments demonstrated that FSINPs enable tumor microenvironment-responsive fluorescence imaging for over 48 h and achieve complete tumor eradication in a 4T1 tumor model without obvious systemic toxicity. This study provides a new strategy for constructing activatable imaging and highly synergistic PTT/CDT/PDT-integrated silk fibroin-based nanomedicines and offers computational chemistry references for their rational design and development.

## 1. Introduction

Malignant tumors represent a major public health issue that seriously threatens human life and health worldwide. Their core pathological features include uncontrolled malignant proliferation, strong invasiveness, and distant metastatic potential, making radical clinical cures extremely challenging [1,2]. Currently, surgical resection, chemotherapy, and radiotherapy remain the mainstream clinical treatment modalities for malignant tumors; however, each of these approaches has certain limitations. Surgery is highly invasive and offers limited efficacy in eliminating occult and metastatic lesions. Chemotherapy and radiotherapy lack therapeutic precision, causing significant systemic toxicity while killing tumor cells. Moreover, long-term treatment tends to induce multidrug resistance in tumor cells, rendering precise and effective tumor eradication difficult [3,4,5]. In recent years, physical stimulus technologies such as light, magnetic fields, and ultrasound have attracted considerable research interest due to their therapeutic accuracy and in situ enhancement effect. These stimuli can precisely act on diseased regions, thereby enhancing the controllability and specificity of therapeutic interventions. For instance, light at specific wavelengths can activate photosensitizers to generate reactive oxygen species (ROS) or thermal effects at the lesion site, consequently destroying cancer cells. Such physical stimulus-triggered therapeutic approaches offer significant advantages, including localized treatment, minimal invasiveness, and reduced systemic toxicity. Although promising, the highly heterogeneous characteristics of the tumor microenvironment, including the acidic extracellular environment, high concentration of glutathione (GSH), and dense extracellular matrix barrier [6,7], severely impede drug penetration and delivery, thereby limiting the efficacy of the therapy [8,9]. Accumulating evidence has demonstrated that multifunctional nanodrug delivery systems can overcome tumor physiological barriers, enhance targeted drug accumulation, and realize synergistic therapy [10,11,12,13,14], representing an effective strategy to enhance these physical stimulus-based therapies.

In the field of nanodrug delivery systems, natural polymeric materials exhibit superior clinical translation potential compared to synthetic polymer-based carriers due to their excellent biocompatibility, tunable degradability, and low immunogenicity [15,16,17]. Among these, silk fibroin nanoparticles (SFNPs) represent an ideal nanoplatform for constructing multifunctional antitumor nanomedicines and have attracted considerable research interest. SFNPs are self-assembled from silk fibroin, a U.S. FDA-approved biomaterial [18,19]. They demonstrate excellent biocompatibility and biodegradability [20], in vivo metabolic pathways, no long-term accumulation toxicity, and minimal immunogenicity. These features overcome the critical biosafety shortcomings of synthetic carriers. Furthermore, SFNPs feature a broad drug-loading spectrum and high loading efficiency. The hydrophobic domains within the SF molecular structure efficiently encapsulate hydrophobic photothermal agents [21], while abundant active groups (e.g., amino and carboxyl groups) along the molecular chains stably bind metal ions via chelation [22], enabling the co-loading and stable delivery of multiple functional components. SFNPs have demonstrated promising application prospects in various antitumor strategies, including chemotherapy [23,24,25], phototherapy [26,27,28], immunotherapy [29], and multimodal combination therapy [30].

Recently, several attempts have been made to construct silk fibroin-based multifunctional phototherapeutic platforms. For example, Yang et al. crystallized MnO_2_ on the surface of SFNPs via biomimetic mineralization to modulate the tumor microenvironment for enhanced phototherapy [26]; Zhang et al. further covalently conjugated Ce6 to MnO_2_-coated SFNPs for enhanced photodynamic therapy [31]; and Li et al. employed a layer-by-layer assembly method to coat Cu_2-X_S nanoparticles with a silk fibroin shell while loading PD-L1 siRNA, forming a multifunctional nanoplatform (CuS-PEI-siRNA-SFNs) that combines cuproptosis induction with photothermal and chemodynamic therapy [32]. Although promising, currently reported silk fibroin-based theranostic platforms still face common challenges, such as complex preparation processes involving multistep procedures, and the underlying molecular mechanisms remain unclear. In particular, the binding modes between the silk fibroin carrier and functional molecules, as well as the interaction mechanisms between metal ions and photosensitizers, have not been thoroughly elucidated.

**Scheme 1:**
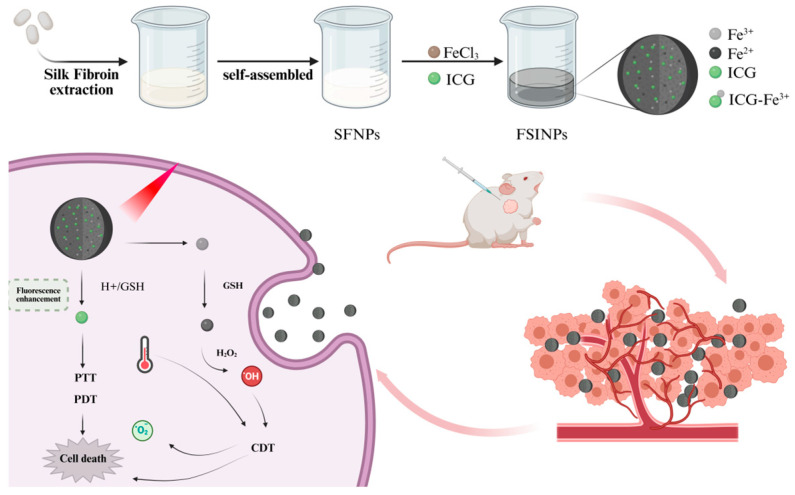
Schematic illustration of the preparation of multifunctional silk fibroin nanoparticles and their antitumor therapeutic mechanism. Scheme 1 was created in BioRender. Xi, Y. (2026) https://BioRender.com/9rx6hb3.

To overcome the aforementioned bottlenecks, we constructed a near-infrared (NIR)-responsive multifunctional silk fibroin-based nanoplatform (termed FSINPs) via a simple self-assembly and adsorption method (Scheme 1). In terms of preparation, this new approach simply enables simultaneous loading of ICG and Fe^3+^ onto SFNPs via sequential stirring adsorption at room temperature, completely eliminating multistep chemical conjugation and biomineralization processes. In terms of functional design, Fe^3+^ endows the nanoplatform with chemodynamic therapy (CDT) capability while synergizing with ICG to achieve a triple-modal combination therapy that integrates photothermal therapy (PTT), photodynamic therapy (PDT), and CDT and leverages the microenvironment-triggered long-term fluorescence enhancement of ICG for sustained tumor imaging. In terms of mechanistic elucidation, we predicted, at the molecular level, the possible hydrogen-bond-driven binding mode between SF and ICG and the electronic structure features of the ICG–Fe^3+^ coordination interaction and proposed corresponding theoretical models. These computational predictions provide a theoretical basis for the rational design of silk fibroin-based nanomedicines.

## 2. Materials and Methods

### 2.1. Materials and Reagents

Silkworm cocoons were obtained from the Northwest Sericulture Base of Shaanxi, China. Sodium carbonate (Na_2_CO_3_), calcium chloride (CaCl_2_), indocyanine green (ICG), and methylene blue (MB) were purchased from Shanghai Macklin Biochemical Co., Ltd., Shanghai, China. 1,3-Diphenylisobenzofuran (DPBF) was purchased from Shanghai Yuanye Bio Technology Co., Ltd., Shanghai, China. Hydrogen peroxide (30% solution, H_2_O_2_) was obtained from Shanghai Lingfeng Chemical Reagent Co., Ltd., Shanghai, China. Anhydrous ethanol was purchased from Shanghai Jizhi Biochemical Technology Co., Ltd., Shanghai, China. Ferric chloride (FeCl_3_) was obtained from Shanghai Aladdin Biochemical Technology Co., Ltd., Shanghai, China. Acetone was purchased from Huzhou Shuanglin Chemical Technology Co., Ltd., Huzhou, China. The calcein-AM/PI cell viability and cytotoxicity assay kit was purchased from Shanghai Beyotime Biotechnology Co., Ltd., Shanghai, China.

### 2.2. Characterization of FSINPs

The morphology of FSINPs was characterized using a transmission electron microscope (TEM) on an FEI Talos F200X microscope (FEI Company, Hillsboro, OR, USA). The elemental composition of the samples was analyzed by X-ray photoelectron spectroscopy (XPS) using an ESCALAB 250Xi photoelectron spectrometer (Thermo Fisher Scientific, Waltham, MA, USA). Ultraviolet–visible (UV-vis) absorption spectra and fluorescence emission spectra were recorded using a SuperMax 3000 microplate reader (Shanghai Flash Spectrum Biological Technology Co., Ltd., Shanghai, China). The generation of ·OH was detected using a Bruker A300 electron paramagnetic resonance (EPR) spectrometer (Bruker, Rheinstetten, Germany). The hydrodynamic diameter, size distribution, and zeta potential of the samples were measured using a Zetasizer Nano ZS (ZEN3600) (Malvern Panalytical, Malvern, UK).

### 2.3. Extraction of Silk Fibroin

Silkworm cocoons were sliced into small, uniform pieces, thoroughly washed with deionized water, dried, and weighed. To remove sericin, the pretreated cocoon pieces were immersed in a 0.5% (*w*/*v*) Na_2_CO_3_ solution at a solid-to-liquid ratio of 1:50 (*w*/*v*). The mixture was heated in a constant-temperature water bath at 100 °C for 60 min under continuous stirring. The degummed silk fibers were then retrieved, rinsed thoroughly with deionized water, and dried at 60 °C to a constant weight. To ensure complete sericin removal, this degumming process was repeated with fresh Na_2_CO_3_ solution at 100 °C for an additional 30 min. Subsequently, the degummed silk fibroin was dissolved in a ternary solvent system of CaCl_2_/C_2_H_5_OH/H_2_O (molar ratio of 1:2:8) in an 80 °C water bath for 4 h. The resulting solution was transferred into a dialysis membrane (molecular weight cut-off, MWCO: 14 kDa) and dialyzed against deionized water for 3 days to eliminate residual ions and solvents. After dialysis, the solution was centrifuged to collect the supernatant, which was pre-frozen at −80 °C overnight and subsequently lyophilized. The obtained purified silk fibroin powder was stored at −20 °C in a sealed container for further experiments.

### 2.4. Preparation of SFNPs

To fabricate SFNPs, an aqueous SF solution (10 mg/mL) was added dropwise to pure acetone at a volume ratio of 1:5 (SF to acetone, *v*/*v*). The mixture was stirred at 600 rpm in a fume hood at room temperature (25 °C) to allow complete evaporation of the acetone. Subsequently, the suspension was transferred to 50 mL centrifuge tubes and centrifuged at 12,000 rpm for 10 min at 4 °C using a high-speed refrigerated centrifuge (Sigma Laborzentrifugen GmbH, Osterode am Harz, Germany). The supernatant was discarded, and the obtained SFNP pellet was collected. The pellet was washed three times with deionized water, with centrifugation repeated under the same conditions after each wash. Finally, the purified SFNPs were redispersed in 10 mL of deionized water and probe-sonicated in an ice bath for 10 min (195 W, pulse cycle of 3 s on/2 s off) to achieve a uniform and stable nanoparticle dispersion.

### 2.5. Preparation of FSINPs

The FSINPs were prepared as follows. First, 2 mL of a 1 mg/mL silk fibroin nanoparticle solution was placed in a beaker, and 100 μL of ICG solution (1 mM) was added dropwise under stirring at room temperature. Subsequently, 300 μL of ferric chloride (FeCl_3_) solution (20 mM) was added to the mixture, and the resulting dispersion was continuously stirred on a magnetic stirrer for 2 h. After the reaction, the mixture was centrifuged at 12,000 rpm for 10 min, and the FSINP precipitate was collected from the bottom of the tube. To remove residual unreacted precursors and impurities, the precipitate was washed three times with deionized water. Finally, the purified FSINP precipitate was redispersed and stored at 4 °C. The encapsulation efficiency (EE) of ICG was calculated using the following formula:

$$EE(\%)=\frac{weight\;of\;loaded\;ICG}{weight\;of\;initially\;added\;ICG}\times 100$$

### 2.6. Computational Study

To investigate the molecular interaction between SF and ICG, molecular docking was performed using AutoDock 4.2.6. The binding energy (kcal/mol) served as the criterion for selecting docked conformations, and the conformation with the lowest binding free energy was selected as the initial structure of the SF–ICG complex. This optimal conformation was subsequently subjected to a 300 ns classical molecular dynamics (MD) simulation using the GROMACS 2023.4 package to further assess the dynamic stability and binding mode of the complex. Global structural stability of the system was evaluated by calculating the root-mean-square deviation (RMSD) of the protein–ligand complex. The residency and binding tightness of the ligand within the binding pocket were characterized by monitoring the dynamic evolution of the distance between the ligand and the center of mass (COM) of the protein. In addition, the temporal variation in the number of hydrogen bonds at the ligand–protein interface during the simulation was statistically analyzed to evaluate the strength and persistence of the interaction between the ligand and the protein. Collectively, these analyses provide a systematic elucidation of the molecular recognition and binding mechanisms underlying the SF–ICG interaction.

Investigation of the binding and fluorescence quenching mechanism of the ICG–Fe^3+^ complex: all calculations in this work were performed using the Gaussian 09 software package employing density functional theory (DFT). Theoretical simulations were utilized to elucidate the fluorescence quenching mechanism of the ICG–Fe^3+^ complex. Geometry optimizations and vibrational frequency analyses were carried out using the PBE1PBE functional with the 6-31G(d) basis set. Solvation effects were taken into account using a water solvent model, and the GD3BJ empirical dispersion correction was included in the calculations.

### 2.7. Characterization of Photothermal Performance

Aliquots of 200 μL of water, SINPs, and FSINPs (with an ICG concentration of 20 μM) were placed in 0.5 mL Eppendorf tubes and irradiated with an 808 nm laser at a power density of 1 W/cm^2^ for 5 min. During irradiation, the temperature was recorded every 10 s using a near-infrared thermal imaging camera (Teledyne FLIR, Wilsonville, OR, USA), and thermal images were captured at 1 min intervals.

FSINP solutions with different concentrations (0, 25, 50, 100, 200, and 400 μg/mL) were prepared. An aliquot of 200 μL of each solution was placed in a 0.5 mL Eppendorf tube and irradiated with an 808 nm laser at a power density of 1 W/cm^2^ for 5 min. Temperature changes were recorded every 10 s using a near-infrared thermal imaging camera. Additionally, FSINPs at a concentration of 200 μg/mL were irradiated with the 808 nm laser at different power densities (0.5, 1.0, and 1.5 W/cm^2^) for 5 min, and the temperature was similarly recorded at 10 s intervals. The photothermal conversion efficiency (η) of FSINPs was calculated using the following formula:

$$\eta =\frac{hs∆T_{FSINPs}-∆T_{water}}{I(1-10^{-A_{808}})}$$

where h is the heat transfer coefficient, s is the irradiation area, TFSINPs and T_water_ represent the temperature changes in FSINPs and water, respectively, I is the laser power (1.0 W/cm^2^), and A_808_ is the absorbance of the sample solution at 808 nm. The product hs can be calculated using the following equation:

$$hs=\frac{mc}{\tau_{s}}$$

where m is the mass of the solution, c is the specific heat capacity of the solvent (4.2 J/g·°C for water), and τ_s_ is the time constant, which can be determined during the cooling period using the following equation:

$$t=-\tau_{s}\times ln(\theta )$$

$$\theta =\frac{T-T_{0}}{T_{max}-T_{0}}$$

where T is the temperature of FSINPs at the cooling time (t) after the light source is turned off, while T_max_ and T_0_ represent the maximum and initial temperatures of FSINPs, respectively.

### 2.8. Detection of ·OH Generation Capability

The capability of generating ·OH and the promoting effect of the photothermal effect on ·OH generation were evaluated using methylene blue (MB) as an indicator. MB can be bleached by ·OH, and the ·OH radicals in the system can be detected by monitoring the change in absorbance at 662 nm. H_2_O_2_ (1 mM) was added to the FSINPs solution (2 mg/mL). The solution was then irradiated with a near-infrared laser (808 nm, 1 W/cm^2^) for 5 min, followed by further incubation for 5 and 10 min. The absorbance of the samples at 662 nm was subsequently measured.

·OH production was quantitatively measured by electron paramagnetic resonance (EPR) spectroscopy using DMPO (5,5-dimethyl-1-pyrroline N-oxide) as a capture agent. DMPO can instantaneously capture ·OH radicals, and the resulting DMPO/·OH adducts can be detected by EPR spectroscopy. FSINPs (1 mg/mL) were mixed with 10 mM H_2_O_2_ and 25 mM DMPO. After 15 min of incubation, the sample solution was analyzed by EPR.

### 2.9. In Vitro PDT Performance Evaluation

^1^O_2_ is a strongly oxidizing reactive oxygen species. The photodynamic properties of FSINPs were investigated using 1,3-diphenylisobenzofuran (DPBF) as a fluorescent probe. Briefly, FSINPs at a concentration of 200 μg/mL were mixed with 10 mM DPBF and then irradiated with a near-infrared laser (808 nm, 1 W/cm^2^) for 5 min. The absorbance of the sample was measured using a SuperMax 3000 microplate reader (Shanghai Flash Spectrum Biological Technology Co., Ltd., Shanghai, China) to evaluate the ^1^O_2_ generation capability of the nanoparticles. Furthermore, to assess the enhancing effect of CDT on PDT, FSINPs were mixed with H_2_O_2_ and DPBF, irradiated with the NIR laser for 5 min, and the absorbance of the samples at 406 nm was subsequently measured.

TEMP was used as a trapping agent: TEMP itself exhibits no EPR signal, but upon reaction with ^1^O_2_, it forms the stable nitroxide radical-TEMPO, which can be detected by EPR. TEMP was added to FSINPs (1 mg/mL), followed by irradiation with an 808 nm near-infrared laser for 5 min. The sample solution was then analyzed by EPR.

### 2.10. In Vitro Cytotoxicity Assay

Murine embryonic fibroblast 3T3 cells and murine breast cancer 4T1 cells were used as models to evaluate the cytotoxicity of different concentrations of FSINPs. The 4T1 mouse breast cancer cell line (STR authenticated) was purchased from Wuhan Procell Life Science & Technology Co., Ltd. (Wuhan, China, Cat# CL-0007) with an equivalent ATCC deposit (CRL-2539; RRID: CVCL_0125). The NIH/3T3 mouse embryonic fibroblast cell line (STR authenticated) was purchased from Wuhan Zishan Biotechnology Co., Ltd. (Wuhan, China, Cat# STCC20005P) with an equivalent ATCC deposit (CRL-1658; RRID: CVCL_0594). Cells were subcultured in RPMI 1640 medium supplemented with 10% fetal bovine serum (FBS) at 37 °C in a 5% CO_2_ atmosphere. Cells in the logarithmic growth phase were used for subsequent experiments.

The cytotoxicity of FSINPs at varying concentrations was assessed using the CCK-8 assay. 3T3 and 4T1 cells were seeded into 96-well plates at a density of 8.0 × 10^3^ cells per well and cultured overnight. The cells were then treated with media containing different concentrations of FSINPs (0, 25, 50, 100, 200, and 400 μg/mL) and incubated for 24 h. After incubation, the supernatant was discarded, and medium containing 10% CCK-8 solution was added to each well. The absorbance at 450 nm was measured using a microplate reader, and cell viability was calculated accordingly.

A combination of the live-cell tracer calcein-AM and the nuclear dead-cell indicator propidium iodide (PI) was used to further evaluate the cytotoxic effects of different nanoparticles on tumor cells. 4T1 cell suspensions were seeded into 96-well plates at a density of 8.0 × 10^3^ cells per well. After an overnight culture to allow cell adhesion, the old medium was removed and replaced with fresh medium containing different nanoparticles, followed by incubation for 6 h. The cells were then irradiated with an 808 nm near-infrared laser (1 W/cm^2^) for 5 min and further incubated for 18 h. After incubation, the staining solution was prepared strictly according to the kit instructions. Then, 200 μL of the mixed staining solution containing calcein-AM and PI was added to each well. After a 30 min incubation in the dark, fluorescence images of live cells (green fluorescence) and dead cells (red fluorescence) were observed and captured using a laser scanning confocal microscope (Ningbo Yongxin Optics Co., Ltd., Ningbo, China).

### 2.11. Animal Experiments

All animal experiments were conducted in compliance with the approval of the Animal Ethics Committee of Zhejiang Sci-Tech University, and all procedures followed the guidelines for animal experiments of Zhejiang Sci-Tech University.

Female BALB/c mice (4–6 weeks) were obtained from Hangzhou Paisiao Biotechnology Co., Ltd. All mice were acclimatized to standard housing conditions for one week prior to the experiment (temperature 22 ± 2 °C, relative humidity 50–60%, 12 h light/dark cycle, with free access to standard rodent chow and sterilized drinking water). The general health status of the animals was monitored daily. To establish the tumor model, 4T1 cells (1 × 10^6^ cells in 0.1 mL PBS) were subcutaneously inoculated into the right forelimb axillary region of female BALB/c mice. When the tumor volume reached approximately 100 mm^3^, the tumor-bearing mice were divided into a free ICG group and an FSINPs group (*n* = 3). Each group received an intratumoral injection at the tumor site, and normal tissue adjacent to the tumor was selected as a subcutaneous injection control. Each site was administered with 100 μL of the corresponding drug (ICG concentration at 1 mg/kg body weight). In vivo fluorescence imaging was performed at 0, 6, 12, 24, and 48 h post-injection to dynamically monitor the biodistribution and metabolism of the drug. After the last imaging, the mice were euthanized by cervical dislocation under isoflurane anesthesia, and the heart, liver, spleen, lungs, kidneys, and tumor tissues were rapidly excised. Following treatment with tissue fixative, ex vivo fluorescence imaging was carried out on each tissue sample to evaluate the differential distribution of ICG among various organs.

Additional tumor-bearing mice (with tumors of approximately 100 mm^3^) were randomly divided into 5 groups (*n* = 5): PBS group, PBS + L group, SINPs + L group, FSINPs group, and FSINPs + L group. Before treatment, the mice were labeled and weighed, and tumor volumes were measured. On days 1, 3, 5, 7, and 9, tumor-bearing mice were intratumorally injected with PBS, SINPs, or FSINPs (ICG content: 1 mg/kg), and the tumors were irradiated with an 808 nm near-infrared laser (1 W/cm^2^) for 5 min. During the treatment, temperature changes at the tumor site were recorded using a near-infrared thermal camera. Mouse body weight and tumor dimensions were measured before each treatment. Animals were humanely euthanized prior to the experimental endpoint if any of the following criteria were met: tumor volume > 2000 mm^3^, severe ulceration, or >20% loss in body weight. On day 14, the mice were euthanized; blood samples were collected from the retroorbital sinus for hematological analysis, and tumors as well as major organs were excised. The excised tumors and organs were fixed in a 4% paraformaldehyde solution, embedded in paraffin, and subjected to hematoxylin and eosin (H&E), Ki67, and TUNEL staining analyses.

### 2.12. Statistical Analysis

Statistical analyses were performed using GraphPad Prism 11 software and Origin 2025b software. All numerical data are presented as mean ± standard deviation (SD). An independent-sample *t*-test was used to determine statistical differences, with the significance threshold set at *p* < 0.05. For differentiation, *, **, and *** represent significance levels of *p* < 0.05, *p* < 0.01, and *p* < 0.001, respectively.

## 3. Results and Discussion

### 3.1. Synthesis and Characterization of FSINPs

**Figure 1:**
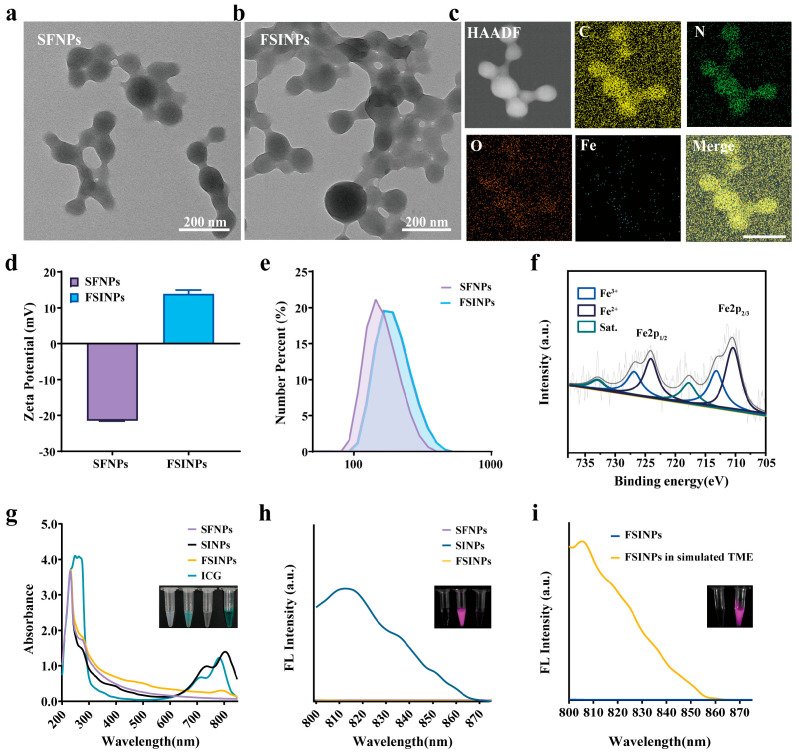
TEM images of (**a**) SFNPs and (**b**) FSINPs. (**c**) Elemental mapping images of FSINPs. (**d**) Zeta potentials of SFNPs and FSINPs. (**e**) Size distribution profiles of SFNPs and FSINPs. (**f**) Fe 2p XPS spectrum of FSINPs. (**g**) UV-vis absorption spectra of different formulations and photographs of SFNPs, SINPs, FSINPs and free ICG. (**h**) Fluorescence spectra and fluorescence images of SFNPs, SINPs, and FSINPs. (**i**) Fluorescence spectra and corresponding fluorescence photographs of FSINPs and FSINPs in a simulated tumor environment. Scale bar = 200 μm.

Transmission electron microscopy characterization (Figure 1a,b) revealed that both SFNPs and FSINPs exhibited relatively uniform spherical-like morphologies. The average particle sizes of SFNPs and FSINPs were determined by measuring at least five monodispersed nanoparticles from TEM images using ImageJ 1.54p software. Statistical analysis showed that the average particle sizes of the two types of nanoparticles were 90.3 ± 19.1 nm and 94.3 ± 13.4 nm, respectively. Energy-dispersive X-ray spectroscopy (EDS) elemental mapping (Figure 1c) demonstrated the homogeneous distribution of C, N, O, and Fe within the nanoparticles, confirming the successful synthesis of FSINPs. As shown in Figure 1d, the zeta potentials of SFNPs and FSINPs were −21.4 ± 0.1 mV and 14.3 ± 1.1 mV, respectively. ICG is negatively charged [33]; thus, the positive shift in zeta potential of the nanoparticles indicates successful chelation of Fe^3+^. Furthermore, DLS measurements showed that the average hydrodynamic diameters of SFNPs and FSINPs were 189.6 ± 1.5 nm and 232.7 ± 2.1 nm, respectively (Figure 1e). The introduction of ICG and Fe^3+^ resulted in a slightly larger particle size for FSINPs compared to SFNPs. The hydrodynamic diameters determined by DLS were larger than those measured by TEM, which can be attributed to the highly swollen state of the nanoparticles in aqueous solution. To evaluate the colloidal stability of FSINPs, DLS was employed to monitor changes in their hydrodynamic diameter under different conditions. As shown in Figure S1, after storage at 4 °C for 48 h, the particle size of FSINPs fluctuated only within a narrow range, with no significant aggregation or degradation observed. Furthermore, after incubation with culture medium containing 10% fetal bovine serum (FBS) at 37 °C for 12 h, the measured hydrodynamic diameter remained stable without an obvious shift or alteration. These findings collectively demonstrate that FSINPs possess good colloidal stability, fulfilling the requirements for subsequent experiments.

To determine the chemical valence states of the metal elements within FSINPs, a comprehensive XPS analysis was performed. As shown in Figure S2, the survey XPS spectrum of FSINPs exhibited characteristic peaks corresponding to C, N, O, and Fe, consistent with the EDS analysis. The Fe 2p peaks of FSINPs appeared at 710.4 eV and 713.2 eV, which are assigned to Fe 2p_3/2_ of Fe (II) and Fe 2p_3/2_ of Fe (III), respectively. The Fe 2p_1/2_ peaks for Fe (II) and Fe (III) were observed at 724.1 eV and 726.9 eV, respectively (Figure 1f), indicating the coexistence of Fe (III) and Fe (II) within the FSINPs [34,35]. The preparation of the nanoparticles was also monitored by UV-vis absorption and fluorescence spectroscopy. As shown in Figure 1g, free ICG in aqueous solution exhibited a distinct characteristic absorption peak at 782 nm. Compared with pristine silk fibroin nanoparticles, ICG-loaded silk fibroin nanoparticles (SINPs) showed a new characteristic absorption peak at 804 nm, which matched the absorption profile of free ICG. The appearance of this characteristic peak verifies the successful incorporation of ICG into SFNPs. After the addition of FeCl_3_ to the SINPs dispersion, the original characteristic absorption peak intensity decreased significantly with a slight blue shift, and the solution color rapidly changed from green to black, indicating the occurrence of a coordination reaction between ICG and Fe^3+^ to form a complex. Fluorescence spectroscopic measurements further supported the above conclusion. Figure 1h shows that SINPs retained the fluorescence characteristics of ICG, and the fluorescence signal of the system was significantly quenched upon the addition of Fe^3+^. As shown in Figure 1i, in a simulated tumor microenvironment, the fluorescence of ICG in FSINPs can be significantly restored, indicating that the nanoplatform has tumor imaging capabilities.

### 3.2. Computational Studies

**Figure 2:**
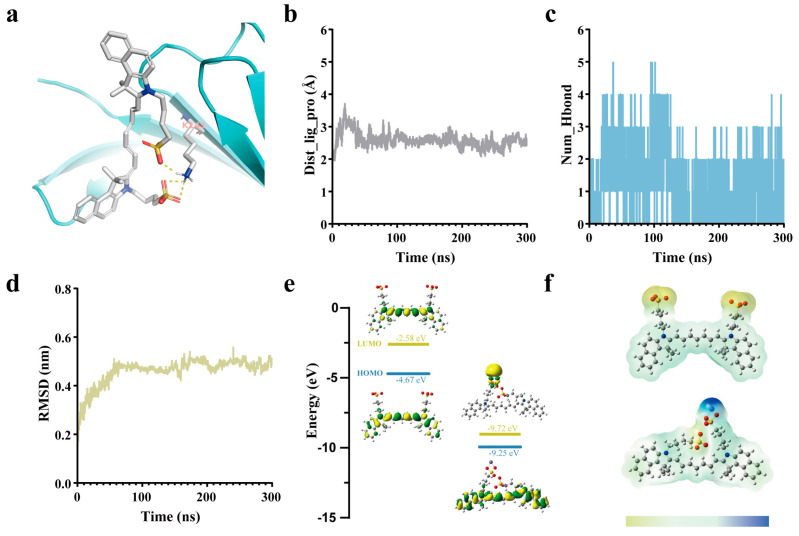
(**a**) Cluster 1 of the ICG-SF complex. (**b**) Distance curve between the ICG molecule and the center of mass of SF. (**c**) Variation curve of the number of hydrogen bonds. (**d**) RMSD curve of the ICG-SF complex. (**e**) Frontier molecular orbitals (HOMO/LUMO) of ICG and the ICG-Fe^3+^ complex. (**f**) Electrostatic potential (ESP) distributions of ICG and the ICG-Fe^3+^ complex, where blue represents regions of positive electrostatic potential and yellow represents regions of negative electrostatic potential.

To investigate the intermolecular interactions involved in the fabrication of the nanodrug, computational simulations were conducted. First, molecular docking between ICG and SF was performed to elucidate the molecular mechanism underlying ICG adsorption by SFNPs. Using AutoDock, the binding modes between ICG and SF were predicted, yielding ten clustered binding conformations. Analysis of binding energies and inhibition constants (Table S1) showed that Conformation 1 had the lowest binding energy (−5.65 kcal/mol) and the smallest inhibition constant (Ki = 71.64 μM) among all conformations, indicating that this conformation possesses the strongest thermodynamic binding affinity and the most favorable theoretical binding activity. Visualization of the binding mode (Figure 2a and Figure S3) revealed that ICG forms extensive hydrogen bonds with multiple amino acid residues in the binding pocket of SF. This hydrogen bond network effectively stabilizes the spatial positioning of ICG within the binding pocket and is a key factor in maintaining stable ligand–protein binding. Among these interactions, hydrogen bonds mediated by lysine and arginine constitute the primary binding sites, while auxiliary hydrogen bonds formed by other polar residues further enhance binding specificity and stability.

To further validate the dynamic stability of the optimal static docking conformation, Conformation 1 was selected for a 300 ns molecular dynamics (MD) simulation. The time evolution of the distance between the ligand and the protein’s center of mass (COM) (Figure 2b) showed that during the initial phase (0–50 ns), the system underwent normal conformational relaxation, with minor fluctuations in the interaction distance. After reaching equilibrium (beyond 50 ns), the ligand–protein distance remained stable within 2.0–3.0 Å, indicating persistently tight binding throughout the simulation. The time course of the number of hydrogen bonds (Figure 2c) revealed that 1–5 hydrogen bonds were consistently maintained between the ligand and the protein over the entire simulation, stabilizing at 1–3 during the equilibrium phase. This confirms the persistence of the hydrogen bond network and its role in stabilizing binding. The RMSD trajectory of the complex (Figure 2d) reflected overall conformational changes in the system. During the first 50 ns, the RMSD increased rapidly, corresponding to a conformational adjustment phase. Subsequently, the RMSD plateaued within 0.45–0.5 nm, exhibiting only minor fluctuations without any apparent sustained drift, indicating that the overall conformation of the ICG–SF complex reached a stable state. Collectively, these results demonstrate stable binding between ICG and SF, with the complex maintaining structural integrity throughout the dynamic process, thereby providing a reliable structural basis for subsequent applications.

In addition to the assembly mechanism of the nanodrug, the optical property of ICG upon metal ion coordination is also critical for its potential bioimaging or therapeutic applications. Therefore, to further elucidate the fluorescence quenching mechanism of ICG upon coordination with Fe^3+^, density functional theory (DFT) calculations were performed to analyze the molecular orbitals and electrostatic potentials of free ICG and the ICG–Fe^3+^ complex. As shown in Figure 2e, the electron cloud of free ICG is primarily localized on its conjugated chain and ring structures, and its characteristic near-infrared absorption arises from the intramolecular π–π transition of the conjugated chain. Upon coordination with Fe^3+^, the highest occupied molecular orbital (HOMO) electron cloud remains concentrated on the ring structure, whereas the electron density in the lowest unoccupied molecular orbital (LUMO) becomes almost completely localized around the Fe^3+^ center. The orbital energy gap is significantly reduced, and the transition type shifts from an intramolecular π–π transition to a ligand-to-metal charge transfer (LMCT). The negative electrostatic potential region of free ICG is mainly concentrated on the sulfonate groups at both termini. After the introduction of Fe^3+^, coordination occurs between the sulfonate groups of ICG and Fe^3+^, and surface electrostatic potential analysis reveals that the negative potential of oxygen atoms is modulated by the positive potential of Fe^3+^ (Figure 2f). Collectively, these computational results indicate that the fluorescence quenching of the ICG–Fe^3+^ complex is attributable to the LMCT process from ICG to Fe^3+^.

Overall, the molecular docking, MD simulations, and DFT calculations support a proposed binding mode between SF and ICG and a ligand-to-metal charge-transfer interpretation of ICG fluorescence quenching by Fe^3+^, providing a comprehensive understanding of the intermolecular interactions during nanodrug fabrication and functional modulation.

### 3.3. Photothermal Performance of FSINPs

**Figure 3:**
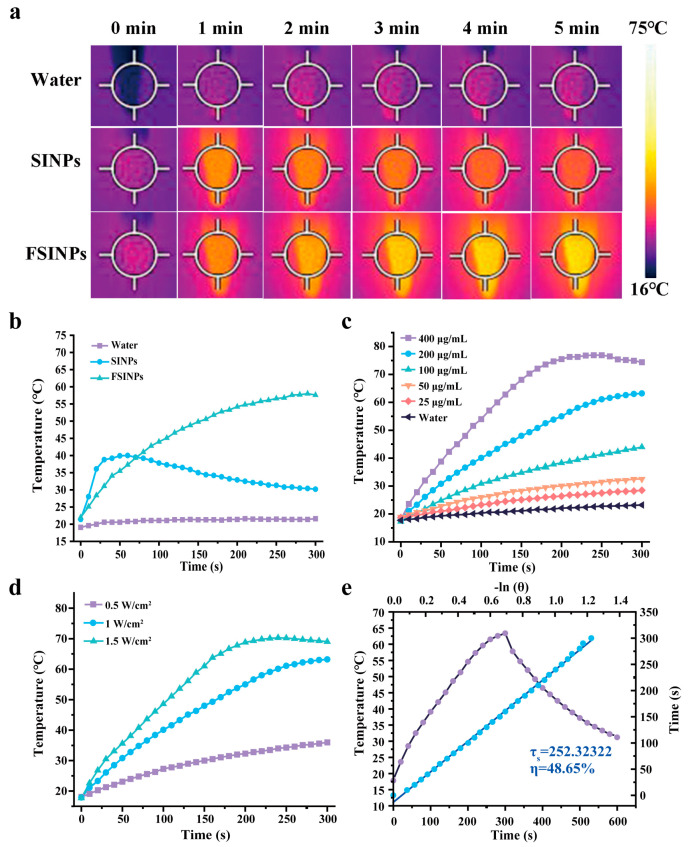
(**a**) Thermal images and (**b**) photothermal heating curves of water, SINPs, and FSINPs under 808 nm NIR irradiation. (**c**) Photothermal heating curves of FSINPs at different concentrations under 808 nm NIR irradiation. (**d**) Photothermal heating curves of FSINPs under NIR irradiation at different power densities. (**e**) Heating and cooling curves of FSINPs and the linear relationship between time (s) and –Ln(θ).

After completing the material characterization and elucidation of the molecular interaction mechanisms, we further investigated the photothermal conversion performance of FSINPs. Figure 3a shows the near-infrared thermal images of different formulations under irradiation at a power density of 1 W/cm^2^. As shown in Figure 3b, the temperature of water remained essentially unchanged upon irradiation, indicating no significant photothermal effect. SINPs exhibited a temperature increase upon NIR irradiation, followed by an obvious temperature decrease after 1 min, which may be attributed to the gradual degradation of ICG in the aqueous environment under NIR exposure. In contrast, after 5 min of NIR irradiation, the temperature of FSINPs increased significantly from 22 °C to 58 °C, far exceeding the temperature rise observed for SINPs. This indicates that the coordination with Fe^3+^ not only enhances the photothermal effect but also improves the photostability of ICG to some extent, enabling FSINPs to maintain a stable temperature elevation under continuous irradiation. Subsequently, we investigated the photothermal heating curves and photothermal conversion efficiency of FSINPs under different power densities or nanoparticle concentrations. As shown in Figure 3c,d, the temperature increase in the FSINPs dispersion gradually intensified with increasing nanoparticle concentration, longer NIR laser irradiation time, and higher laser power density. After 5 min of continuous irradiation with an 808 nm laser at a power density of 1 W/cm^2^, the temperature of the FSINPs dispersion at a concentration of 200 μg/mL reached 63.2 °C, fully demonstrating its efficient photothermal conversion response. Furthermore, to quantitatively characterize the photothermal conversion capability of this nanosystem, the temperature–time cooling curve during the natural cooling phase was analyzed, and the photothermal conversion efficiency (η) was derived using the corresponding calculation formula. The obtained photothermal conversion efficiency was approximately 48.7% (Figure 3e); this value markedly exceeds that of most reported ICG-based nanoplatforms [36], and the enhancement is ascribed to the Fe^3+^-coordination-induced fluorescence quenching. After light absorption by ICG, the energy that would normally be emitted as fluorescence is channeled into non-radiative relaxation via Fe^3+^ coordination, thus dissipating as heat.

### 3.4. Detection of Hydroxyl Radical Generation Capability

**Figure 4:**
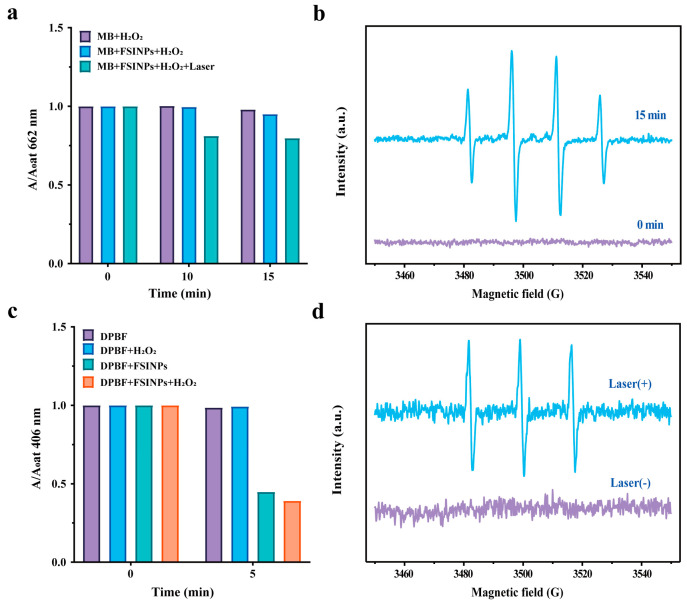
(**a**) MB-based detection of the ·OH generation capability of FSINPs: changes in absorbance at 662 nm after 10 min and 15 min of incubation under different treatments. (**b**) EPR spectra of FSINPs for ·OH detection before and after incubation with H_2_O_2_. (**c**) DPBF-based detection of ^1^O_2_ generation: changes in absorbance at 406 nm of FSINPs and control groups before and after NIR irradiation. (**d**) EPR spectra of FSINPs for ^1^O_2_ detection before and after NIR irradiation.

In addition to photothermal performance, the chemodynamic activity of FSINPs was assessed by detecting hydroxyl radical generation. In this study, methylene blue (MB) was employed to investigate the ·OH generation capability of the nanoparticles and the synergistic enhancement effect of PTT on CDT. As shown in Figure 4a, when MB was treated with H_2_O_2_ alone, its absorbance showed a slight decrease within 15 min, indicating that H_2_O_2_ alone has extremely low degradation efficiency toward MB. When FSINPs and H_2_O_2_ were both added to the system, the degradation rates of MB at 662 nm were 0.4% and 5.0% after 10 and 15 min of incubation, respectively. Upon additional NIR laser irradiation of the same system, the degradation rates rose to 18.9% and 20.2% at the respective time points. These results further indicate that FSINPs can mediate the degradation of MB, and the introduction of NIR-induced photothermal effects further accelerates this process, suggesting a synergistic promotional effect of PTT on CDT. Subsequently, EPR spectroscopy was employed to further verify the generation of ·OH. DMPO undergoes a rapid and specific addition reaction with ·OH to form a relatively stable spin adduct, DMPO OH. This converts the hydroxyl radical, which has a lifetime on the order of nanoseconds, into a stable adduct with a lifetime of several minutes, thereby enabling detection by an EPR spectrometer. As shown in Figure 4b, no distinct EPR signal was detected for FSINPs at 0 min, indicating that the material does not generate free radicals in its initial state and possesses good physiological stability. After 15 min of incubation in a simulated tumor microenvironment, a characteristic ·OH signal emerged, demonstrating that FSINPs can efficiently generate ·OH through the Fenton reaction [37]. This finding provides direct evidence of the free radical mechanism underlying the subsequent induction of tumor cell death.

### 3.5. In Vitro PDT Performance Evaluation

ICG loaded in FSINPs is a classic near-infrared photosensitizer. Under 808 nm NIR irradiation, ICG absorbs photon energy and undergoes a transition from the ground state (S_0_) to the excited singlet state (S_1_), followed by intersystem crossing (ISC) to the excited triplet state (T_1_). The excited triplet ICG then transfers energy to ground state oxygen (^3^O_2_) in the surrounding environment, converting it into highly toxic ^1^O_2_. ^1^O_2_ can directly oxidize and damage biomacromolecules in tumor cells, including lipids, proteins, and DNA, ultimately inducing cell death and achieving PDT [38]. DPBF exhibits a highly specific reaction with singlet oxygen and is commonly used as an indicator of singlet oxygen. Upon reaction with singlet oxygen, its fluorescence is quenched, or its absorbance peak decreases, thereby enabling the detection of singlet oxygen. As shown in Figure 4c, after 5 min of NIR irradiation, the absorbance at 406 nm of the FSINPs group decreased significantly compared with the DPBF group. Upon addition of H_2_O_2_ to the system, the decrease became even more pronounced, while the DPBF group showed no significant change upon H_2_O_2_ addition. This enhanced effect may be attributed to the decomposition of H_2_O_2_ catalyzed by Fe^3+^ during CDT, which produces O_2_ and provides more substrate for PDT [39], thereby alleviating tumor hypoxia and enhancing therapeutic efficacy.

^1^O_2_ is captured by the spin trap TEMP to form a stable nitroxide radical-TEMPO adduct, which generates a characteristic triplet EPR signal that can be detected. As shown in Figure 4d, no distinct EPR signal was detected for FSINPs in the absence of laser irradiation, indicating that the material does not generate ^1^O_2_ under dark conditions and exhibits good physiological stability. Upon NIR laser irradiation, a characteristic TEMP ^1^O_2_ peak appeared, directly demonstrating that FSINPs can efficiently generate highly toxic ^1^O_2_ through the ICG-mediated photodynamic effect.

### 3.6. Cytotoxicity and In Vitro PTT/PDT/CDT Therapeutic Efficacy

**Figure 5:**
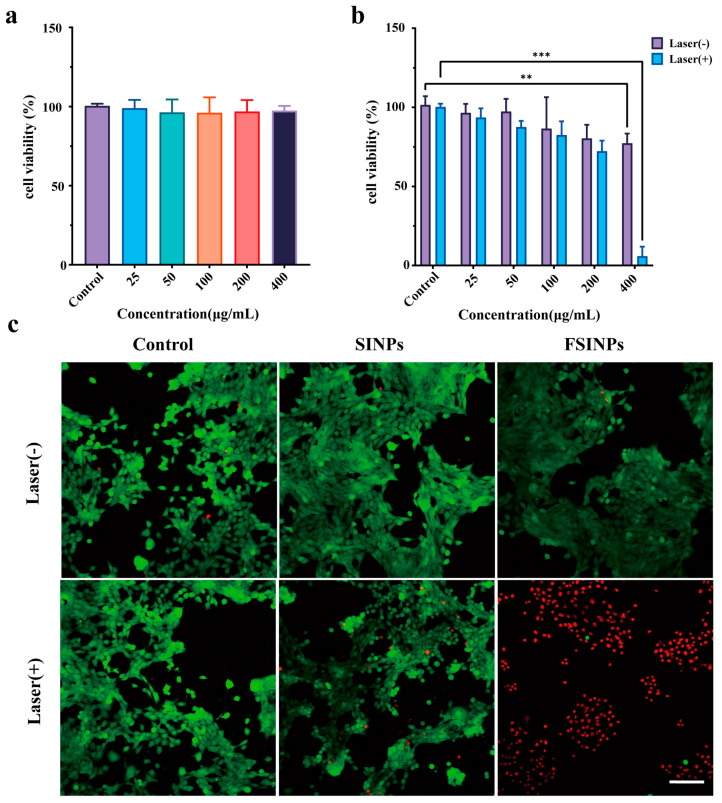
(**a**) Viability of 3T3 cells after incubation with different concentrations of FSINPs for 24 h (*n* = 5). (**b**) Viability of 4T1 cells treated with different concentrations of FSINPs or FSINPs + laser (*n* = 5). (**c**) Fluorescence images of 4T1 cells co-stained with calcein-AM and PI after different treatments. Scale bar = 100 μm. Using independent sample *t* test, ** = *p* < 0.01, *** = *p* < 0.001.

Based on the promising ROS generation and photothermal properties, we proceeded to evaluate the in vitro antitumor efficacy of FSINPs. As shown in Figure 5a, the viability of 3T3 normal cells incubated with FSINPs for 24 h remained above 90% across all tested concentrations (0–400 μg/mL), with no significant difference from the untreated control group. This indicates that FSINPs do not exert obvious adverse effects on normal cells. In Figure 5b, the viability of 4T1 cells exhibited a clear concentration-dependent decrease. Compared with the blank control group, the viability of 4T1 cells progressively decreased with increasing nanoparticle concentration. At a nanoparticle concentration of 400 μg/mL, the cell viability was 75.9%, indicating that the nanoparticles exhibit moderate cytotoxicity toward tumor cells. Further mechanistic analysis suggests that this cytotoxic effect is primarily attributed to the Fe element in the nanoparticles: Fe reacts with endogenous H_2_O_2_ in cells to continuously generate toxic ·OH, which, as highly reactive oxygen species, induces cell death, ultimately leading to a concentration-dependent decrease in cell viability [40]. Notably, FSINPs exhibited obvious selective cytotoxicity toward tumor cells over normal cells. This selectivity is likely attributable to the higher oxidative stress level and endogenous H_2_O_2_ content in tumor cells compared to normal cells, which provide more abundant substrates for the Fe^3+^-mediated Fenton reaction, thereby preferentially exerting cytotoxic effects. Moreover, upon combination with 808 nm NIR irradiation, the cell viability decreased dramatically with increasing material concentration. At 400 μg/mL, the cell viability dropped to merely 5.9%, demonstrating a significant photo-activatable antitumor effect. This synergistic enhancement is not a simple additive effect but originates from a positive feedback loop among PTT, CDT, and PDT: the laser-triggered hyperthermia not only directly ablates tumor cells but also accelerates the kinetics of the Fenton reaction through temperature elevation, substantially increasing the generation rate of ·OH; meanwhile, the O_2_ generated during CDT provides sufficient oxygen substrate for PDT, effectively alleviating the hypoxia-induced suppression of photodynamic therapy and further amplifying the overall ROS-mediated killing effect. The live/dead cell staining assay further confirmed that the FSINP-mediated synergistic therapy achieved the highest cytotoxicity among all treatment groups. Laser scanning confocal microscopy observations (Figure 5c) revealed that the majority of cells in the control group exhibited live cell-specific green fluorescence, indicating a well-maintained viable state. In contrast, the experimental group treated with FSINPs in combination with NIR irradiation displayed abundant dead cell-specific red fluorescence.

### 3.7. In Vivo Tumor Fluorescence Imaging and NIR Thermal Imaging

**Figure 6:**
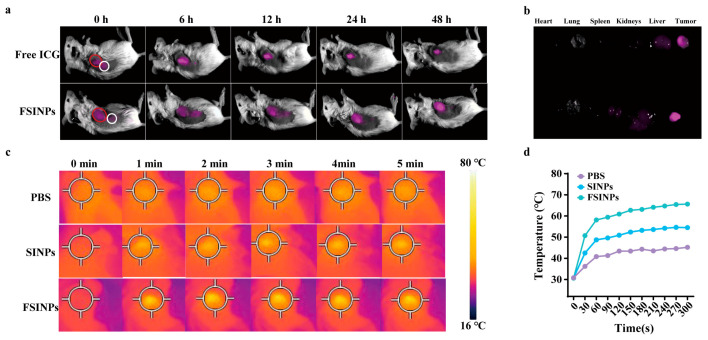
(**a**) In vivo fluorescence imaging of mice injected with free ICG or FSINPs. The red circle indicates intratumoral injection at the tumor site, and the white circle indicates subcutaneous injection in the adjacent normal tissue. (**b**) Ex vivo fluorescence imaging of tumors and major organs. (**c**) Near-infrared thermal images and (**d**) tumor temperature change curves of tumor-bearing mice injected with PBS, SINPs, or FSINPs followed by irradiation with an 808 nm laser (1 W/cm^2^).

In light of the near-infrared fluorescence characteristics of ICG, the in vivo fluorescence imaging performance of the nanoparticles was evaluated. As shown in Figure 6a, the fluorescence signal at the tumor site in the intratumorally injected free ICG group peaked at 6 h post-administration and gradually decayed thereafter. In contrast, the fluorescence signal in the subcutaneously injected free ICG group completely disappeared by 6 h. Strikingly, the tumor fluorescence signal in the FSINP-treated group exhibited a continuous increase over the 48 h observation period, while the subcutaneously injected FSINP group showed a peak signal at 6 h followed by a decline, indicating prolonged local retention and time-dependent fluorescence enhancement behavior of this nanoformulation at the tumor site. In addition, the fluorescence in the tumor tissues of the FSINP-treated group was also significantly stronger than that of the ICG group (Figure 6b). These results demonstrate that the FSINPs can be retained at the tumor site and exhibit excellent tumor near-infrared imaging performance.

Figure 6c,d show the thermal images and corresponding temperature rise curves of the tumor site in tumor-bearing mice following intratumoral injection of PBS, SINPs, or FSINPs and subsequent irradiation with an 808 nm NIR laser for 5 min. Detailed temperature data revealed that in the PBS control group, the tumor temperature increased only slightly from 30.8 °C to 45.2 °C after laser irradiation. The SINP-treated group exhibited a more pronounced temperature elevation, reaching 54.6 °C at the endpoint. Notably, the FSINP-treated group showed the most remarkable photothermal effect, with the tumor temperature rising to 65.6 °C. The differences in temperature increase among the three groups were highly significant. These results fully demonstrate that FSINPs retain excellent photothermal conversion performance even in the complex physiological environment in vivo, indicating strong potential for antitumor applications.

### 3.8. In Vivo Antitumor Efficacy

**Figure 7:**
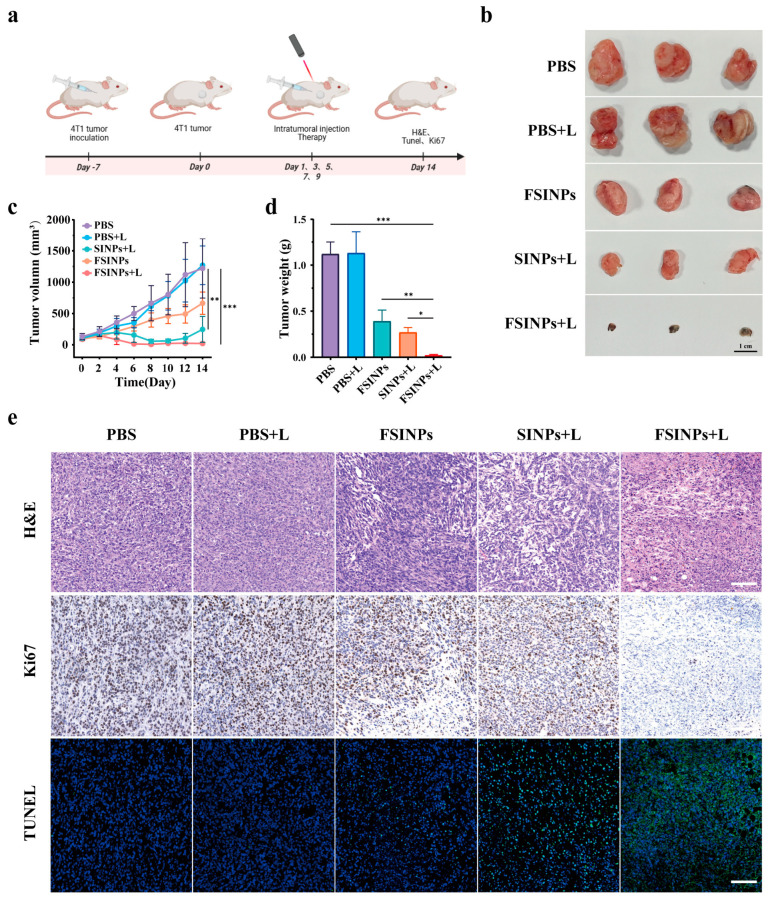
(**a**) Schematic illustration of tumor treatment with FSINPs, created in BioRender. Xi, Y. (2026) https://BioRender.com/a7p25oc. (**b**) Tumor volume growth curves. (**c**) Tumor weights (*n* = 5) and (**d**) photographs of excised tumors after treatment (*n* = 5). (**e**) H&E, immunohistochemical Ki67, and TUNEL staining images of tumor sections from each group. Scale bar = 100 μm. Using independent sample *t* test, * = *p* < 0.05, ** = *p* < 0.01, *** = *p* < 0.001.

Given the excellent cell-killing capability of the nanoparticles in vitro, we further investigated the combined therapeutic efficacy of FSINPs against 4T1 tumors. Figure 7a presents a schematic illustration of the experimental procedure.

As shown in Figure 7b, the mice were randomly divided into five groups: PBS, PBS + L, SINPs + L, FSINPs, and FSINPs + L. After the treatment period, the PBS and PBS + L groups exhibited rapid tumor growth, indicating that NIR irradiation alone did not significantly inhibit tumor progression. Compared with the PBS group, the FSINPs group showed a slower increase in tumor volume, which may be attributed to the release of iron ions at the tumor site, inducing the CDT effect and thereby suppressing tumor growth. In the SINPs + L group, tumor volume gradually decreased after the third treatment administration but increased again after the fifth treatment, suggesting that PTT alone exerts an obvious antitumor effect, yet recurrence is highly likely after treatment cessation. Notably, in the FSINPs + L group, tumor volume began to decrease after the second treatment and remained unchanged after the fifth administration. This demonstrates that the synergistic effect enhances therapeutic efficacy, achieving more thorough tumor elimination and inhibiting the proliferation of residual tumor cells, thereby preventing tumor regrowth after the five treatment cycles.

The ex vivo tumor weights (Figure 7c) and tumor photographs (Figure 7d) of each group collectively confirmed the superior antitumor performance of FSINPs. To further validate the antitumor efficacy of FSINPs, the excised tumor samples were subjected to H&E staining, terminal deoxynucleotidyl transferase-mediated dUTP nick-end labeling (TUNEL) staining, and immunohistochemical Ki67 staining (Figure 7e). H&E staining revealed necrotic lesions of varying severity in tumor tissues from the FSINPs, SINPs + L, and FSINPs + L groups, compared with the PBS and NIR-alone (PBS + L) control groups. The most extensive necrosis and the severest damage were observed in the FSINPs + L combination treatment group. Ki67 immunohistochemical staining further corroborated the intergroup differences in therapeutic efficacy at the level of cell proliferation. The PBS and PBS + L control groups exhibited abundant brown-stained Ki67-positive proliferating cells throughout the tumor tissues. Although the number of Ki67-positive cells was reduced in the FSINPs monotherapy group and the SINPs + L photoirradiation group, proliferative activity remained relatively high. In contrast, the FSINPs + L group showed a marked decrease in Ki67-positive cells, demonstrating the strongest tumor proliferation inhibitory effect. TUNEL apoptosis fluorescence staining was used to assess the level of programmed cell death in tumor cells across the groups. In the PBS and PBS + L control groups, almost no green apoptotic fluorescence was detected. The FSINPs monotherapy group exhibited only sporadic and weak apoptotic fluorescence signals, while the SINPs + L group showed a clear increase in apoptotic signals. Notably, the FSINPs combined with NIR irradiation group displayed extensive bright green apoptotic fluorescence throughout the field of view, with the degree of apoptosis far exceeding that of all other groups. Histological staining results revealed that FSINPs monotherapy achieves moderate antitumor efficacy, and near-infrared light synergistically strengthens its effects on inducing tumor apoptosis and suppressing cell proliferation. Collectively, these data validate the robust in vivo antitumor performance of FSINPs. Its therapeutic core lies in multimodal synergistic effects to eliminate tumor cells and restrain proliferation, rendering FSINPs a promising candidate for tumor treatment.

### 3.9. In Vivo Biosafety Evaluation of FSINPs

**Figure 8:**
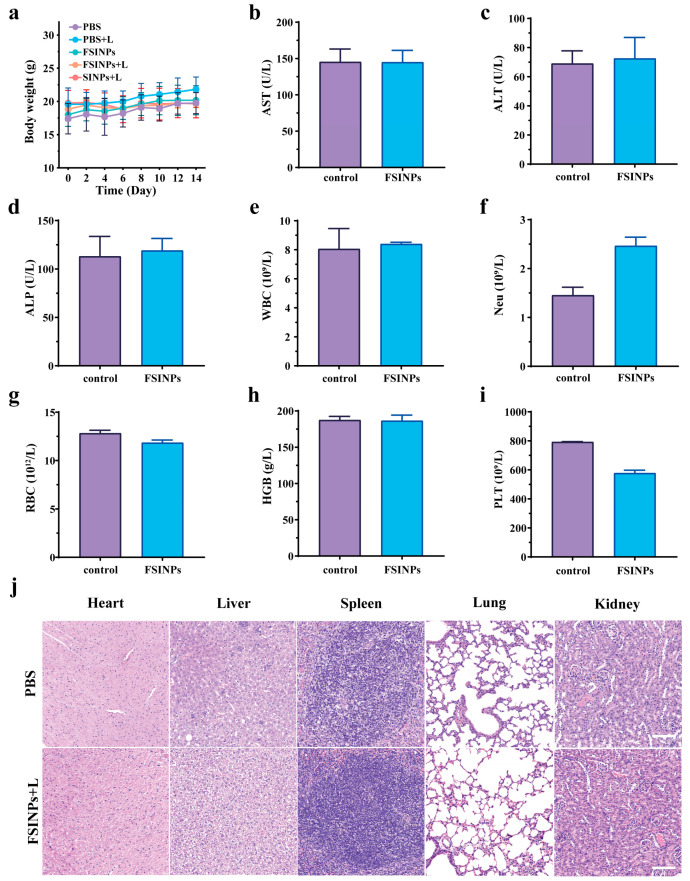
(**a**) Body-weight changes (*n* = 5); (**b**–**d**) aspartate aminotransferase (AST), alanine aminotransferase (ALT), and alkaline phosphatase (ALP) (*n* = 3); (**e**–**i**) white blood cells (WBCs), neutrophils (Neu), red blood cells (RBCs), hemoglobin (HGB), and platelets (PLT) (*n* = 3); and (**j**) representative H&E images of major organs after PBS or FSINPs + laser treatment. Scale bar = 100 μm.

A comprehensive evaluation of the biosafety of FSINPs was conducted. As shown in Figure 8a–d, no significant changes in body weight or serum biochemical parameters were observed among the treatment groups after administration. The results of hematological analysis (Figure 8e–i) indicated that all measured parameters in mice treated with FSINPs were within the normal range. Although Neu levels (Figure 8f) showed a mild increase and PLT levels (Figure 8i) exhibited a slight decrease compared with the control group, both parameters remained within the normal reference ranges, suggesting that FSINPs did not induce hematological toxicity. The observed variations may be attributed to normal individual biological fluctuations or a subtle physiological adaptation following nanoparticle exposure. In particular, a minor elevation in neutrophils can occur as part of a transient immune response or inflammatory adaptation, while slight fluctuations in platelet counts may result from differences in platelet production, activation, or distribution. Importantly, no abnormal changes were observed in other hematological parameters, and the overall blood profile remained stable, indicating good hemocompatibility of FSINPs. As shown in Figure 8j and Figure S4, histopathological examination of major organs (heart, liver, spleen, lungs, and kidneys) was performed using H&E staining. No significant pathological abnormalities were observed in any of the major organs following FSINP treatment. These results demonstrate that FSINPs possess excellent biocompatibility and that their outstanding antitumor efficacy arises from the synergistic effects rather than nonspecific damage to normal tissues.

## 4. Conclusions

In summary, this study successfully developed an NIR-responsive silk fibroin-based nanoplatform for long-term tumor imaging and synergistic PTT/CDT/PDT. FSINPs were constructed via a facile self-assembly and chemisorption strategy. The iron component within FSINPs triggers Fenton reactions with the high concentration of H_2_O_2_ in the tumor microenvironment, continuously generating highly reactive ·OH that efficiently induces tumor cell death and exerts specific cytotoxic effects. The photothermal conversion efficiency of the nanoparticles reaches as high as 48.7%, and the local hyperthermia generated under NIR irradiation not only directly ablates tumor cells but also effectively enhances the efficiency of CDT. Furthermore, CDT promotes the efficacy of PDT to a certain extent. The FSINP-mediated synergistic PTT/CDT/PDT not only significantly improves antitumor efficacy and overcomes the limitations of single-modality therapies but also, by virtue of the excellent biocompatibility of the silk fibroin carrier, minimizes off-target damage to normal tissues. Beyond the therapeutic efficiency, FSINPs also exhibit a fluorescence recovery effect in the tumor microenvironment, enabling tumor microenvironment-responsive fluorescence enhancement. Moreover, by integrating multiple computational simulation approaches, this study elucidated at the molecular level the binding mode between indocyanine green and silk fibroin, as well as the fluorescence quenching mechanism based on ligand-to-metal charge transfer, revealing the intercomponent interaction mechanisms that have been rarely reported previously. Collectively, this study provides an innovative and practical strategy for the development of high-performance and safe anti-cancer nanomedicines.

## Data Availability

The original contributions presented in this study are included in the article/Supplementary Materials. Further inquiries can be directed to the corresponding authors.

## Supplementary Materials

The following supporting information can be downloaded at https://www.mdpi.com/article/10.3390/ma19153359/s1.

## Abbreviations

The following abbreviations are used in this manuscript:

CDT	chemodynamic therapy
DLS	dynamic light scattering
DMSO	dimethyl sulfoxide
DPBF	1,3-diphenylisobenzofuran
EPR	electron paramagnetic resonance
FBS	fetal bovine serum
FSINPs	Fe-doped ICG-loaded silk fibroin nanoparticles
GSH	Glutathione
H_2_O_2_	hydrogen peroxide
ICG	indocyanine green
MB	methylene blue
NIR	near-infrared
PDT	photodynamic therapy
PTT	photothermal therapy
ROS	reactive oxygen species
SF	silk fibroin
SFNPs	silk fibroin nanoparticles
SINPs	ICG-loaded silk fibroin nanoparticles
TEM	transmission electron microscopy
TEMP	2,2,6,6-tetramethylpiperidine
TEMPO	2,2,6,6-tetramethylpiperidine-1-oxyl
XPS	X-ray photoelectron spectroscopy
