# Supplementary material for: Multifunctional Near-Infrared-Responsive Silk Fibroin Nanomedicine for Tumor Treatment and Imaging
Source: Materials (Basel). 2026 Aug 6;19(15):3359. doi: 10.3390/ma19153359 (PMC13468336; doi:10.3390/ma19153359)
Supplement: Supplementary file 1 [file materials-19-03359-s001.zip › materials-4432300-supplementary.pdf]

Supporting Information

Fabrication and Antitumor Activity of Near-Infrared Responsive Multifunctional Silk Fibroin Nanoparticles

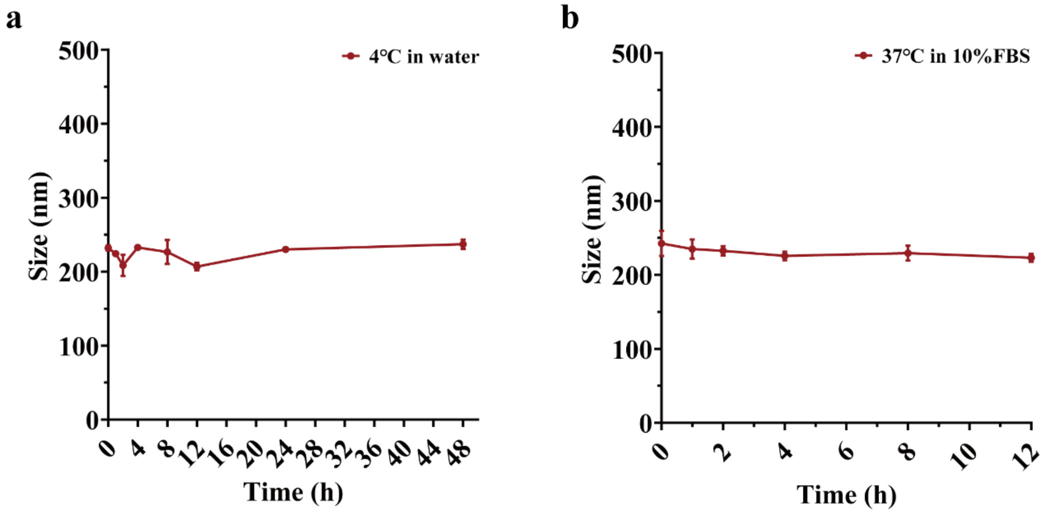

Figure S1. Time-resolved DLS monitoring of hydrodynamic particle size to evaluate the colloidal stability of FSINPs. (a) Temporal size variation of FSINPs upon 12 h incubation with 10% fetal bovine serum-containing medium at 37 °C. (b) Long-term size fluctuation of FSINPs under 48 h static storage in pure water at 4 °C.

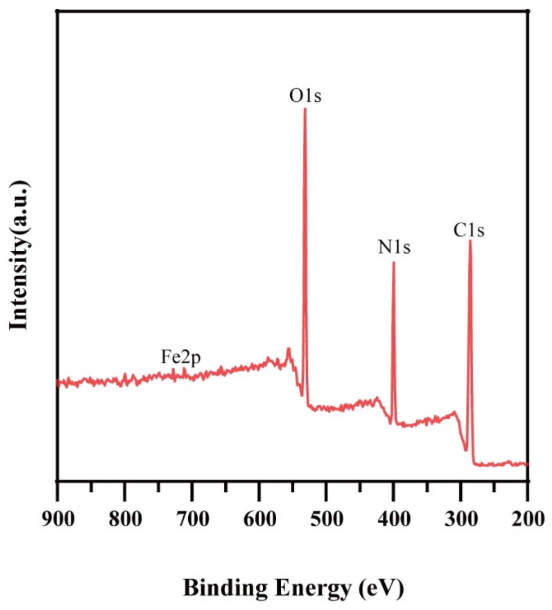

Figure S2. XPS spectrum of FSINPs.

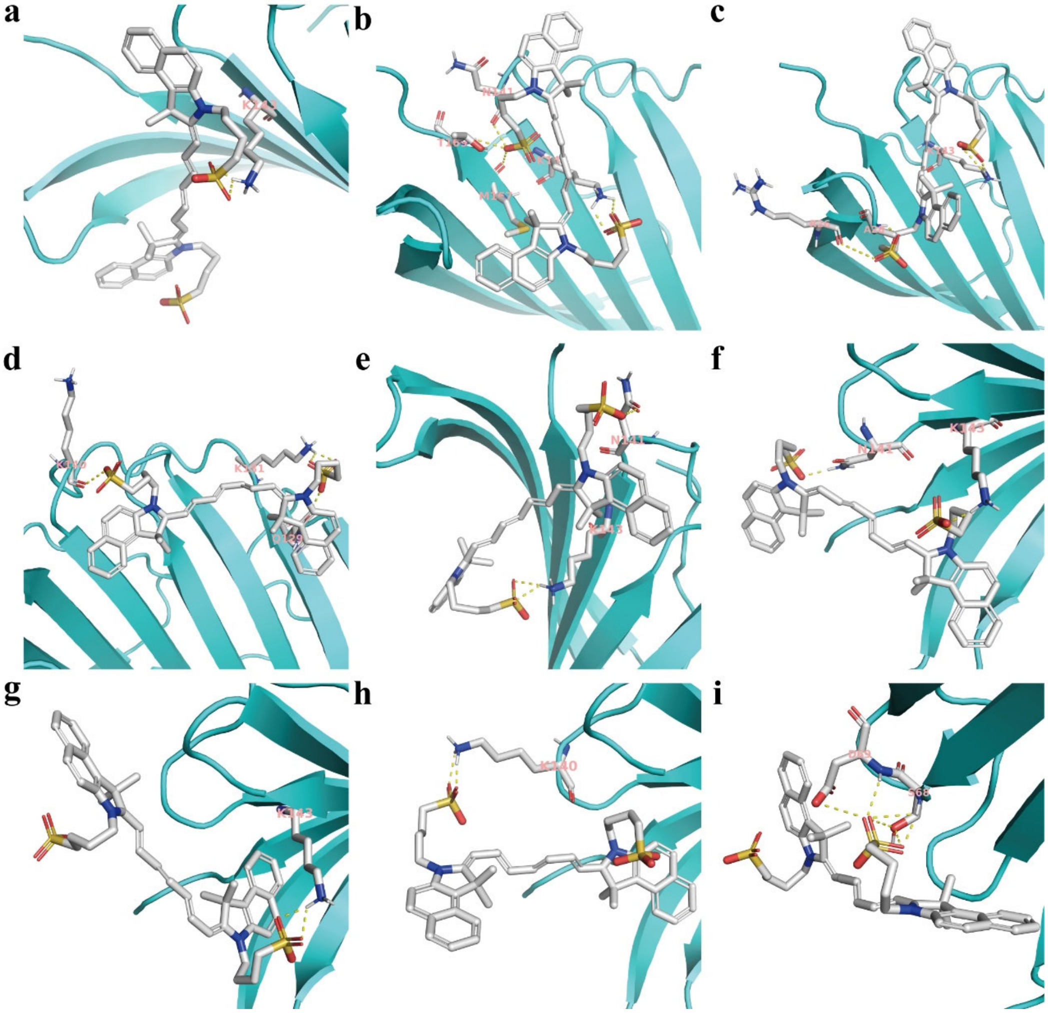

Figure S3. The nine representative conformations of the ICG–SF complex obtained from docking are shown in panels (a-i).

Table S1. Cluster analysis results of the docking conformations**.**

Cluster	Binding Energy (kcal/mol)	Ki	Intermolecular Energy	vdW+Hbond +desolv	Electrostatic	Internal Energy	Torsional Energy	RMSD (Å)
1	-5.65	71.64 μM	-9.83	-8.24	-1.59	-2.58	4.18	26.448
2	-4.93	243.05 μM	-9.11	-8.02	-1.09	-3.4	4.18	23.32
3	-4.93	244.78 μM	-9.1	-8.09	-1.02	-3.26	4.18	24.982
4	-4.89	258.44 μM	-9.07	-8.44	-0.63	-3.69	4.18	25.503
5	-4.21	827.06 μM	-8.38	-7.4	-0.98	-3.3	4.18	28.575
6	-4.01	1.15 mM	-8.19	-7.46	-0.73	-3.22	4.18	23.709
7	-3.91	1.37 mM	-8.08	-7.33	-0.75	-3.92	4.18	29.32
8	-3.83	1.55 mM	-8.01	-6.37	-1.64	-3.05	4.18	29.277
9	-3.56	2.45 mM	-7.74	-6.47	-1.26	-3.58	4.18	31.086
10	-3.32	3.71 mM	-7.49	-8.45	0.96	-4.19	4.18	30.627

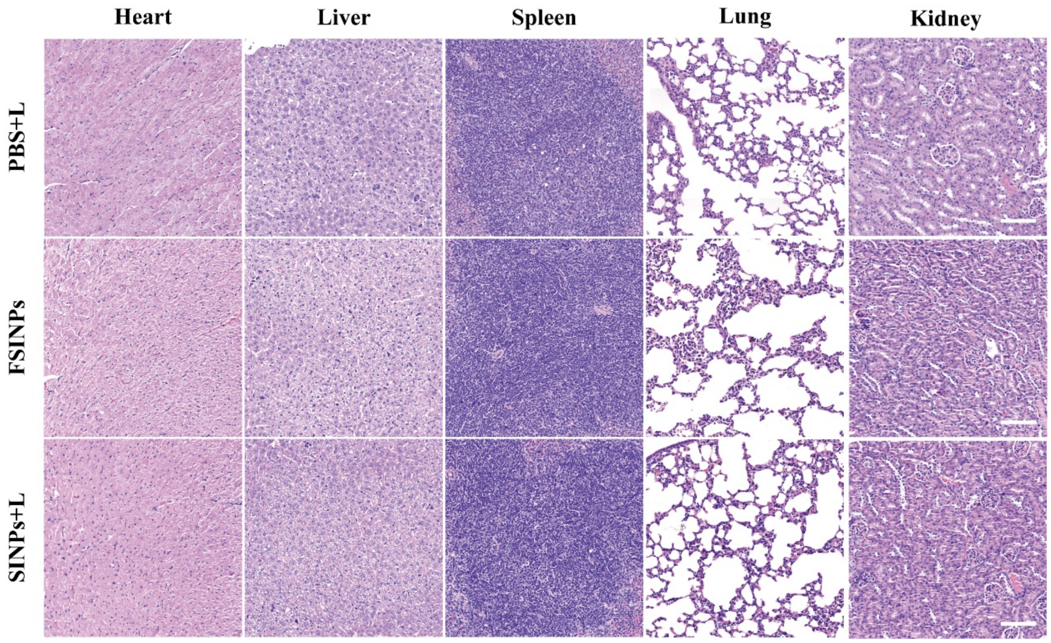

Figure S4. Representative H&E staining images of major organs after treatment of PBS+L, FSINPs, and SINPs+L
